# Electrochemotherapy: technological advancements for efficient electroporation-based treatment of internal tumors

**DOI:** 10.1007/s11517-012-0991-8

**Published:** 2012-11-21

**Authors:** D. Miklavčič, G. Serša, E. Brecelj, J. Gehl, D. Soden, G. Bianchi, P. Ruggieri, C. R. Rossi, L. G. Campana, T. Jarm

**Affiliations:** 1 Faculty of Electrical Engineering, University of Ljubljana, Ljubljana, Slovenia; 2 Institute of Oncology Ljubljana, Ljubljana, Slovenia; 3 Center for Experimental Drug and Gene Electrotransfer, Department of Oncology, Copenhagen University Hospital Herlev, Copenhagen, Denmark; 4 Cork Cancer Research Centre, Mercy University Hospital and Leslie C. Quick Jnr Laboratory, Grenville Place, Cork, Ireland; 5 Oncological Department, Istituto Ortopedico Rizzoli, Bologna, Italy; 6 Sarcoma and Melanoma Unit, Veneto Region Oncology Research Institute (IOV-IRCCS), Padua, Italy

**Keywords:** Electroporation, Electrochemotherapy, Cancer treatment, Treatment planning

## Abstract

Electrochemotherapy, a combination of high voltage electric pulses and of an anticancer drug, has been demonstrated to be highly effective in treatment of cutaneous and subcutaneous tumors. Unique properties of electrochemotherapy (e.g., high specificity for targeting cancer cells, high degree of localization of treatment effect, capacity for preserving the innate immune response and the structure of the extracellular matrix) are facilitating its wide spread in the clinics. Due to high effectiveness of electrochemotherapy in treatment of cutaneous and subcutaneous tumors regardless of histological origin, there are now attempts to extend its use to treatment of internal tumors. To advance the applicability of electrochemotherapy to treatment of internal solid tumors, new technological developments are needed that will enable treatment of these tumors in daily clinical practice. New electrodes through which electric pulses are delivered to target tissue need to be designed with the aim to access target tissue anywhere in the body. To increase the probability of complete tumor eradication, the electrodes have to be accurately positioned, first to provide an adequate extent of electroporation of all tumor cells and second not to damage critical healthy tissue or organs in its vicinity. This can be achieved by image guided insertion of electrodes that will enable accurate positioning of the electrodes in combination with patient-specific numerical treatment planning or using a predefined geometry of electrodes. In order to be able to use electrochemotherapy safely for treatment of internal tumors located in relative proximity of the heart (e.g., in case of liver metastases), the treatment must be performed without interfering with the heart’s electrical activity. We describe recent technological advances, which allow treatment of liver and bone metastases, soft tissue sarcomas, brain tumors, and colorectal and esophageal tumors. The first clinical experiences in these novel application areas of electrochemotherapy are also described.

## Electroporation

When a cell is exposed to an external electric field, a transmembrane voltage is induced on the cell membrane and when it exceeds a “threshold” value, electroporation of the cellular membrane occurs, i.e., hydrophilic pores form in the membrane and the flow of molecules in and outward of the cell substantially increases [32]. Although the threshold for electroporation depends on numerous physical (e.g., cell size and shape), biological (e.g., cytoskeleton structure and membrane composition) and electrical parameters (e.g., amplitude, duration and number of pulses, pulse repetition frequency), it is possible to achieve electroporation in vitro and in vivo regardless of the cell or tissue type [69]. This universal effectiveness of electroporation has made it a popular technique for loading cells with substances that are otherwise not possible or difficult to transfer into the cells. Today, electroporation is used in numerous biotechnological and clinical applications. In biotechnology it is used for microbial deactivation of liquid food like fruit and vegetable juices and prolongation of their shelf time, without using high temperatures that are usually needed for pasteurization of juices [51, 72], or for enhancing extraction of substances from cells, such as sugar from sugar beet [60]. In the medical field, however, applications such as gene transfection for gene therapy [16] and DNA vaccination [61], and electrochemotherapy for treatment of skin melanoma metastasis, skin recurrences of breast cancer, head and neck tumors and other histological types have been reported [27, 41, 42, 50, 64].

## Electrochemotherapy

### Basic description

**Fig. 1:**
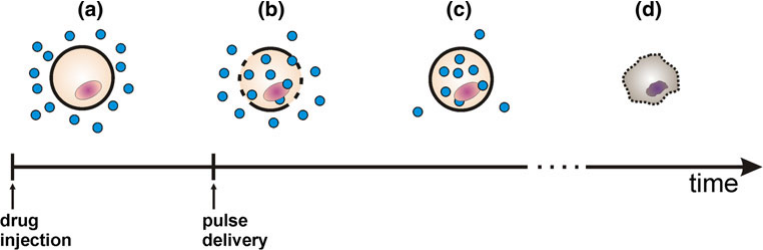
Basic concept of electrochemotherapy: **a** after injection drug surrounds the cells; **b** formation of pores after pulse delivery, drug enters the cells; **c** membrane resealing, drug entrapped inside the cells; **d** drug kills the cells

In electrochemotherapy (ECT), short and intense electric pulses are delivered to tumor nodules in vivo using appropriate electrodes to transiently electroporate the membranes of cancer cells and thus enable direct access to the cytosol (Fig. 1) [45, 67]. Under these conditions, a cytotoxic agent such as bleomycin, which is hydrophilic and lacks efficient uptake mechanisms, can readily enter the cells. Since the increased uptake is only at the site of application of electric pulses, ECT is essentially a local treatment. Once inside, bleomycin acts like an enzyme, creating several single and double strand DNA breaks. Only the cells undergoing mitosis are affected by bleomycin [47, 53]. Cisplatin is another cytotoxic drug successfully used in ECT [63, 68]. The tissue volume in which electroporation occurs is determined by the electric field distribution, which can be controlled by appropriate type and number of electrodes and their positioning, and by choice of electric pulse amplitude at a given number and duration of pulses [44]. The cytotoxic drugs used clinically, predominantly affect actively dividing cancer cells and to a lesser extent the largely non-dividing population of normal cells in the surrounding tissue [25, 41]. These two properties assure a high therapeutic index of ECT.

The following advantages demonstrated in treatment of cutaneous and subcutaneous tumor nodules make ECT an exceptionally appealing cancer treatment [41]:

- High response rate in local tumor control: complete response is achieved in 59.4 % (regardless of histological origin) and partial response in additional 24.7 % of the treated nodules after a single-session ECT [39];
- Further increase in response rate of the tumors that are not responding completely to a single-session ECT can be achieved by repetitive ECT at several weeks’ intervals [10, 59];
- Limited effect on healthy tissues: the specificity and low cumulative doses of chemotherapeutic drug used in ECT spare the surrounding normal cells and tissues, preserving the function of all organs;
- Treatment of tumor margin: not only the volume of the tumor is treated, but also the safety margin, thus eliminating possible infiltrating tumor cells;
- Preservation of immune response: ECT does not cause protein denaturation, therefore, the antigens, if present at the site, are not destroyed and may elicit an immune response on remaining tumor cells (if any) after ECT;
- Effective in previously treated areas, either after surgery or radiotherapy;
- Favorable toxicity profile: in clinical use no immediate or late side effects are associated with ECT;
- Advantageous cost-benefit ratio of the technology involved and the drugs used, in particular bleomycin: no large investment is required [13];
- Possible performance on an outpatient basis: even when general anesthesia is needed, due to the short duration of the treatment (less than 1 h), only short hospitalization is required.

### Summary of clinical electrochemotherapy experiences

Currently, ECT is used in treatment of cutaneous and subcutaneous tumors of different histological types with response rate of 84.1 % and long lasting complete responses rate of 59.5 % [39]. The treatment is predominantly used with palliative intent for melanoma metastases but also other cutaneous nodules. After initial studies performed in Europe (France and Slovenia) and USA starting in early 90s [4, 19, 49], ECT is now being used predominantly in Europe, where there are currently 16 countries with at least one clinical center offering it to its patients (Table 1). In 2011, 1,500 patients have been treated with ECT, and as registered by several patient databases, ECT is currently being used in over 100 clinical centers (Table 2). Several reviews have elaborated on clinical uses of ECT, reporting also on clinical results and benefits of ECT [65, 71]. The development of ECT can be divided into periods before and after ESOPE project (European Standard Operating Procedures for Electrochemotherapy—an EU-funded project). This project has provided the standard operating procedures for ECT using Cliniporator (a CE marked electric pulse generator for clinical use, developed within a preceding EU-funded project of the same title) [48] and based on the results of a prospective multicenter clinical study on single treatment of cutaneous nodules of different histology, using ECT with bleomycin or cisplatin, given intratumorally or intravenously [41]. The results of the study have confirmed previously published results [62, 66] and set the stage for dissemination of the technology in treatment of cutaneous tumor nodules in Europe.

**Table 1:** Electrochemotherapy use since 2006 when standard operating procedures were established and published [48], geographical distribution

Country	Number of clinical centers
Italy	31
Spain	5
Germany	31
Austria	1
Sweden	4
Great Britain/Ireland	6
Greece	5
Portugal	1
Poland	1
The Netherlands	1
Hungary	1
Slovenia	1
France	6
Denmark	1
Belgium	1
Lithuania	1
Research	12
Total	109

Data provided by IGEA S.p.A., Carpi, Italy

**Table 2:** Electrochemotherapy use since 2006 when standard operating procedures were established and published [48], number of patients treated

Years	Number of patients treated	Number of clinical centers
2006	130	
2007	300	37
2008	600	53
2009	800	55
2010	1,000	83
2011	1,500	92
2012*	3,000	160

Data provided by IGEA S.p.A., Carpi, Italy

* Figures for 2012 are based on prediction

Based on encouraging results obtained after ESOPE study, the treatment is now acknowledged and funded by national insurance agencies in several countries [70]. Therefore, in many of the listed countries in Table 1, ECT is now in routine clinical practice, predominantly for treatment of cutaneous metastases of melanoma, and is also included in EU guidelines for treatment of melanoma [70]. After the cutaneous melanoma metastases, the next major step for use of ECT seems to be in treatment of chest wall recurrences of breast cancer, for which the clinical benefit has already been demonstrated [64] and also the results of the first phase II clinical study have been published [43, 64].

## Tissue electroporation and technological developments

Electroporation of cellular membrane occurs when a transmembrane voltage is induced across the membrane by exposure to external electric fields. Since the induced transmembrane potential is proportional to the magnitude of the electric field, the electric field distribution in tissue is the most important determinant of successful electroporation-based treatments [44]. Numerical modeling is currently the only efficient way to predict electric field distribution in biological tissues, as these are characterized by inherently non-homogeneous, nonlinear and, in some cases, anisotropic dielectric properties [57]. State-of-the-art numerical models that incorporate changes in electrical conductivity of tissues because of electroporation have helped explain how tissues beneath the *stratum corneum* can be electroporated by electric pulses [56]. Also, numerical modeling has been used to demonstrate the importance of ensuring good surface contact between the electrodes and tissue in case of plate electrodes [14], and the importance of the depth of insertion in case of needle electrodes [15]. Both these factors can be crucial for complete tumor eradication. It was also shown how a combination of numerical modeling and optimization methods could be used for treatment planning of electroporation-based treatments, by determining the optimal electrode position and electric pulse amplitude [74]. This concept has already been successfully used in a clinical case [20, 46] and is being further developed. The approach using numerical modeling is a topic of intensive ongoing research and validation [55].

The uptake of the chemotherapeutic drug after application of electroporation pulses by tumor cells is another crucial determinant of successful electrochemotherapy (ECT). Uptake of the drug depends on its local availability—which is related to intratumoral drug distribution, and the level of membrane permeabilization which depends on local electric field. As tumors are inhomogeneous, both in terms of drug distribution as well as in electrical conductivity, one should not assume both will always or easily be achieved. The only way to assure that the drug has reached the target tissue is by injecting the drug prior to pulse delivery. In this way the maximum extracellular drug concentration in the tumor can be achieved before delivering pulses, but because of intratumoral heterogeneity this in itself, does not guarantee that sufficient drug concentration has been reached in all parts of the tumor. The optimal time between drug injection and pulse delivery depends on the drug and route of its administration. Currently, in clinical studies the Standard Operating Procedures (SOP) are being followed in which, based on experience and literature available at that time for cisplatin and bleomycin, an intratumoral injection is to be followed immediately by application of pulses, whereas for bleomycin given systemically an 8-min waiting time is suggested before application of pulses [48]. In case of systemic injection, if the pulses were applied first, then due to the vascular lock effect [30] the drug would have hindered access to the tumor; intratumoral injection, however, should work just the same as long as it falls within the time before pores reseal, i.e., within the time of increased membrane permeability. The impact of different orders and timings of drug delivery and application of pulses were tested in vivo [11, 63]. In case of systemic application of the drug the optimal “treatment window” for application of pulses extends considerably over the 8-min interval (up to 28 min after drug injection according to the SOPs). According to some studies in which concentration of bleomycin was measured at different intervals after systemic injection the “treatment window” for application of pulses may be even longer, up to 1 h or more [23]. One important benefit of this wide “treatment window”, which is considered a well-established fact, is that it allows several tumors to be treated by application of pulses after a single systemic injection of the drug.

The electroporation of tissue at a given number and duration of pulses depends on local electric field distribution [58], and this in turn is a function of the electrode geometry and configuration/positioning, tissue anatomy and tissue dielectric properties. Therefore, it is in principle possible to treat target tissue, e.g., solid tumors, in any location in the body, provided that appropriate electrodes and an image-guided assistance for accurate positioning of the electrodes relative to the target tissue are available. The development of this methodology started with identification of possible deep-seated target tumors and locations. Large tumors (>3 cm in diameter) being located in soft tissue like colorectal metastases in liver, soft tissue sarcomas located deep below the skin and bone metastases were initially identified as possible target tissues. Furthermore, tumors in gastric tube were identified as possible target for ECT, as well as brain tumors. All these cases are routinely subject to radical and often mutilating surgical intervention with poor prognosis and in many cases leading to poor quality of life [18].

ECT is of course not the only ablation technique available for local treatment of liver and other internal tumors. Radiofrequency ablation for example is one of the well-established ones [6]. It complements surgical resection of tumors, and can also be done percutaneously under ultrasound guidance. However, due to heat sink effect, radiofrequency ablation is not recommended in the vicinity of major hepatic vessels and leads to frequent tumor recurrences [29, 54]. Therefore, ECT provides a non-thermal approach for treatment of tumors in such locations, as was demonstrated in the first described case of successful treatment of un-resectable tumor located within major hepatic vessels [20]. In order to bring ECT into broader clinical application, however, further clinical studies comparing its effectiveness with radiofrequency ablation will be needed.

**Fig. 2:**
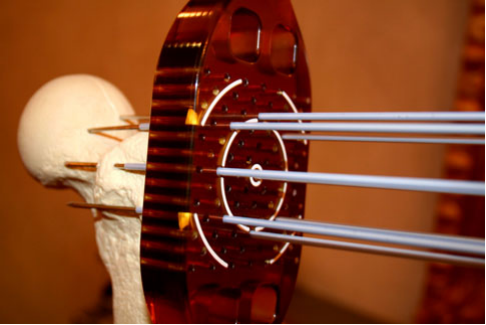
Electrodes with trocar tip for direct insertion by drilling into the bone or bone metastasis. The tip is bare allowing electrical contact whereas the rest of the electrode is insulated. The holder provides parallelism of electrodes and facilitates insertion of electrodes at predefined distances to each other

The new generation of the Cliniporator device (Cliniporator VITAE, Igea, Carpi, Italy) has been designed for treatment of large volumes of tissue. This machine uses up to six independent needle-shaped electrodes that can be positioned independently to treat the tumor regardless of its shape and size [5] but also electrodes with fixed geometry can be used [35]. This pulse generator delivers electric pulses with standard duration of 100 μs with voltage and current amplitudes of up to 3,000 V and 50 A, respectively. Having up to six independent electrodes and being able to deliver electric pulses between any two of these electrodes allows treatment of tumors of roughly up to 128 cm^3^ (8 cm × 4 cm × 4 cm) or even larger. Two different types of needle-like electrodes were developed: with 1.2-mm diameter and sharp tip for insertion into soft tissue, and with 1.8-mm diameter and trocar tip for direct insertion into the bone and bone metastasis by means of a drilling machine (Fig. 2). Both types of electrodes are 20-cm long and covered with insulation except at the tip, where 2, 3 or 4 cm of the bare electrode length is exposed.

**Fig. 3:**
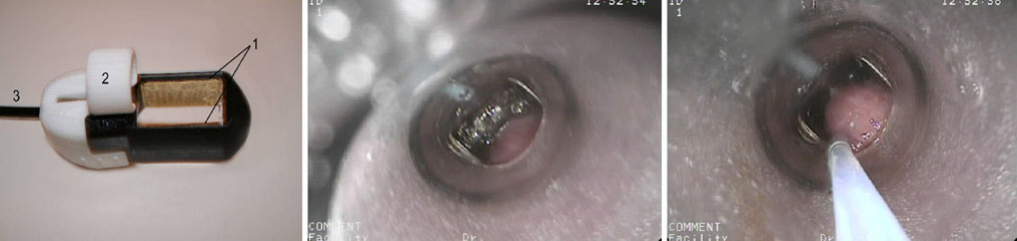
Image of an EndoVE prototype (*left*) that is then attached to the endoscope head: *1* the electrodes, *2* holder for attachment to the head of an endoscope, *3* cable connection. *Middle* and *right* view through the endoscope head of esophageal tissue being drawn into the device chamber. On the *right* the tissue under pressure has entered the electrode chamber and is being injected with a drug via the endoscope biopsy port. The two electrodes are placed in parallel on the walls inside the chamber. Electric pulses are delivered to the tissue captured between the two electrodes

For application of ECT in gastric tube, an endoscopic type of electrodes with fixed geometry has been developed (EndoVE device; Fig. 3). Two plate electrodes of 1 cm × 2.6 cm surface area with a distance of 1 cm in between are mounted on two opposite sides of a small chamber. The tissue is sucked into this chamber by means of under pressure and when brought in between the two plate electrodes the electric pulses are applied. If tumor is larger than the chamber, the chamber is repositioned and the next section of the tumor is sucked into the chamber and electroporated. Such steps are repeated until the whole tumor mass is sequentially electroporated. The guidance of the electrodes is through an endoscopic channel as the chamber with the electrodes is mounted onto the endoscope.

**Fig. 4:**
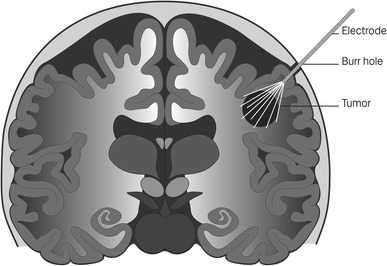
Electrodes inserted into brain through a drilled hole

For brain tumors and metastases an expandable probe (consisting of several electrodes) has been developed, which is introduced through a single burr hole through the scull and then electrodes are released, so that they spread out and penetrate the target tissue (Fig. 4) in a way similar to that used in radiofrequency ablation (RFA) treatment. In a comprehensive project, a novel electrode for electroporation-based drug and gene delivery to soft tissue was designed and produced [26]. The initial goal was to use the electrode for treatment of primary and secondary brain tumors, but other soft tissue tumors could also be envisaged as potential targets. The basic concept is to have one insertion channel, and then to be able to expand the electrodes to encompass the entire target area. Predefined geometry has been carefully designed and optimized [35], and preclinical testing has shown the validity of the principle [3].

### Early clinical experience of treating internal tumors: liver metastases

Colorectal cancer has become the most frequent cancer in Europe (excluding the non-melanoma skin cancer) with annual estimated incidence of more than 430,000 newly diagnosed cases and is the second leading cause of cancer-related mortality with more than 210,000 deaths annually [21]. At the diagnosis, 14.5 % of patients have detectable colorectal liver metastases [24] and during the course of the disease 14.5 % of the patients develop liver metastases after primary surgical treatment [40]. Radical surgical resection of liver metastases remains the only chance of cure with more than 50 % 5-year survival [2]. Unfortunately, only minority of patients with colorectal liver metastases have a disease that is potentially resectable. Disseminated disease is usually treated by systemic chemotherapy and biological drugs. Many regional therapies are developed for non-resectable liver metastases but with only limited success.

**Fig. 5:**
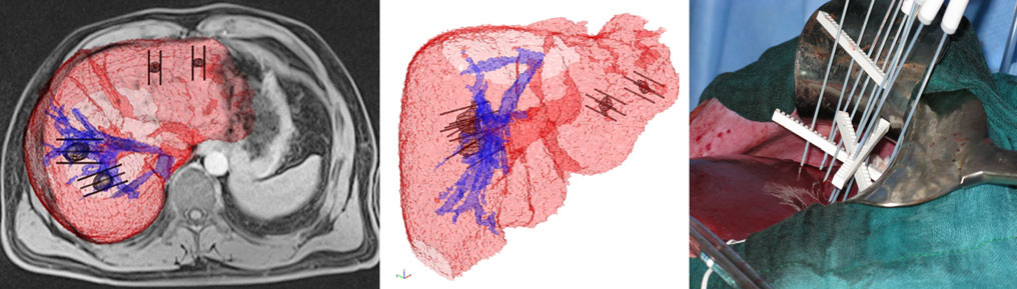
Specific patient treatment plan is prepared based on medical images. The plan is prepared using numerical modeling and provides number of electrodes, their placement with respect to the tumor tissue, and amplitudes of pulses to be delivered and between which electrodes. It serves the surgeon to insert the electrodes into and around target tissue

At the Institute of Oncology Ljubljana, a phase I and II clinical study on treatment of liver metastases by ECT is currently underway (NCT01264952) with the aim to evaluate toxicity and effectiveness of ECT with bleomycin in treatment of liver metastases of colorectal cancer. Two groups of patients are being included in the study: patients with multiple liver metastases of colorectal cancer planned for the two-step hepatectomy procedure and patients with a solitary metastasis in between inferior vena cava and the trunks of the main hepatic veins. The first group includes the patients with multiple, bilobar and/or enlarged colorectal metastases (mostly synchronous, however metachronous cases are included as well) which exceed the limits for safe, one-step surgical procedure and where the two-step hepatectomy procedure would be performed in any case. In these patients, the region of liver containing tumors treated with ECT during the first surgery is removed during the second surgery about 6 weeks later. The second group includes patients with colorectal metastases which are found to be non-resectable and untreatable with other ablation techniques. The treatment of colorectal liver metastases is performed in a similar way as for cutaneous nodules, using the same dosage of bleomycin (15 mg/m^2^) and delivering the electric pulses between the long needle electrodes of variable geometry using Cliniporator VITAE (Igea, Carpi, Italy). ECT is performed according to the pre-prepared treatment plan, which determines the electrode placement, pairs of electrodes that need to be activated and the amplitudes of electric pulses to be delivered. The treatment plan is based on CT/MRI scans of the treatment region and on individualized numerical optimization of electroporation parameters (Fig. 5). Electric pulses are delivered according to the ESOPE protocol within a treatment window of 8–28 min after systemic injection of bleomycin [20]. To maximize safety of the patient, the delivery of electric pulses is synchronized with electric activity of the heart to prevent the pulses from being delivered during the vulnerable period of the ventricles, which otherwise could interfere with functioning of the heart due to relative proximity of the treatment region and the heart [17, 37].

In the ongoing clinical study until now ECT has been performed on ten patients. The preliminary data indicate that the treatment is safe, even in difficult to treat locations; no adverse heart-related effects were recorded [38]. The treatment has proved to be safe also when tumors were located in between major hepatic veins. The first described patient is now 30 month after the application of ECT and subsequent removal of the tumor, without evidence of the disease [20]. In this case, the histological analysis of the liver region resected 6 weeks after ECT demonstrated a complete destruction of the tumor treated by ECT. Furthermore, preliminary evaluation of data of all ten treated patients demonstrated evidence of radiological changes in all ECT-treated metastases that resemble changes typically observed after RFA. The response to ECT was evaluated as complete in 50 % and as partial in the remaining 50 % of treated metastases.

Some of the patients included in the study were scheduled for the two-stage hepatectomy operation; during the first operation, ECT was performed on some of the metastases, while the other metastases located in the part of the liver to be preserved were treated by surgery or RFA and the right portal vein was ligated when indicated. Portal vein occlusion induces hypertrophy of the future remnant liver, allowing curative hepatectomy during the second operation. In such cases, histological analysis is possible for evaluation of histological changes in the treated lesions, especially with respect to the other metastases that were in the same hepatic lobe not treated with ECT. Preliminary histological analysis indicated that ECT induced progressive degenerative changes in all ECT-treated metastases; namely significantly less viable tumor tissue was found remaining in ECT-treated metastases than in non-ECT-treated metastases (*p* < 0.001).

However, the protocol for ECT of liver metastases needs further refinement with respect to the electrode insertion guidance and real-time positioning using ultrasound imaging. Currently the electrodes are not sufficiently visible under ultrasound. Treatment planning also needs further development, to be of help also during the procedure in the operating room, allowing modifications of electrode positioning, according to the clinical situation encountered in situ and providing immediate adjustments of electrical parameters for effective treatment. The preliminary data furthermore demonstrated that with development of the ECT technique for liver metastases ECT could be beneficially used in treatment of all liver tumors. The advantages of ECT over other treatment modalities appear predominantly in difficult-to-treat locations, where standard ablation techniques would not have been effective or possible. However, for superficial metastases that can be treated with electrodes with fixed geometry, no individualized treatment plan is needed. Using fixed geometry electrodes ECT can be performed very quickly on several metastases in a short period of time.

### Early clinical experience of treating internal tumors: bone metastases

The incidence of bone metastases in Europe is about 1,100,000 per year [12]. Metastatic disease affecting bones is a major cause of decreased quality of life in patients with cancer and its impact on patients’ quality of life is significant because of pain, pathological fractures, spinal cord compression and reduction of movement and performance status.

In addition to systemic treatments (chemotherapy) for disease control, pain relief is one of the most challenging and significant problems; in fact, when surgery is not indicated (unresectable bones such as pelvis or in case of disseminated disease), the use of narcotics is not always effective and the use of local ablative techniques such as RFA or selective embolization can be as well ineffective. Radiotherapy can be considered the standard treatment; however, external beam radiation can be inefficient in 20 to 30 % of patients [9, 31] and a “limit of dose” can be reached for each metastases thus preventing further treatment.

After preclinical studies that demonstrated bone osteogenic activity and bone hardness to be preserved after ECT [22], a phase I–II clinical trial is ongoing at Istituto Ortopedico Rizzoli (Bologna, Italy) in order to asses safety and feasibility of ECT on bone metastases. After primary staging (bone scan, CT/MRI, etc.), ten patients affected by bone metastases of the appendicular skeleton (no path fracture, no previous local treatment in the past three months and maximum tumor volume of 100 cm^3^) were treated with ECT. An electric pulse generator (Cliniporator VITAE, Igea, Carpi, Italy) was used to deliver electroporation pulses to the metastases via bone needle electrodes surrounding the metastases. Bleomycin was administrated intravenously (15 mg/m^2^) 8 min before the electric pulses were delivered, thus allowing building up bleomycin concentration in the tumor. Nine more patients with pelvic or sacral metastases were treated “out of protocol” and under CT guidance because the tumor was deep seated. Two spine metastases were also treated during open surgery, but results are not available yet. The procedure was usually carried out in general anesthesia and lasted about 60 min with the patient discharged the day after treatment. ECT response was evaluated radiographically by either MRI or CT imaging 4 and 8 weeks after ECT. Pain relief was evaluated with VAS score and EORTC QLQ-C30 questionnaire [1].

**Fig. 6:**
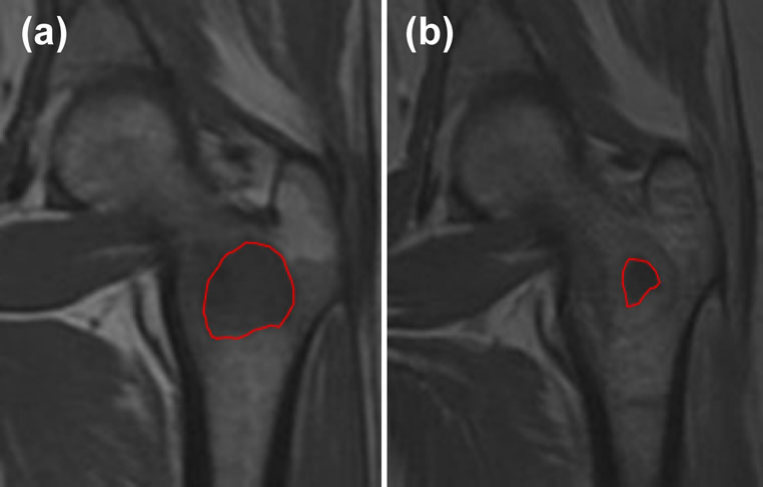
Breast metastatic carcinoma of the proximal femur at presentation (**a**) and reduction of the metastasis 5 weeks after electrochemotherapy (**b**). *Red line* a guide to the eye (colour figure online)

Early results show pain relief in 56 % of the patients with quality of life improvement [7]. In some cases radiographic response was also observed with necrosis of the metastatic lesion (Fig. 6). Although these preliminary clinical results are still to be confirmed by a longer follow-up and larger cohort of patients, early results are encouraging and continuous improvements of the techniques including efforts towards pretreatment planning are ongoing.

### Early clinical experience of treating internal tumors: soft tissue sarcoma

Soft tissue sarcomas (STS) are a heterogeneous group of rare mesenchymal neoplasms that account for roughly 1 % of all adult malignancies. Currently, more than 50 histotypes of STS have been identified. The variability in patients’ clinical outcome underscores the heterogeneity of the biological aggressiveness of these tumors, the therapeutic management of which remains one of the most challenging tasks for oncologists. STS can occur anywhere in the body, but most originate in the extremities (about 60 %). Radical resection can be functionally mutilating for patients and technically demanding for surgeons. Moreover, most STS are characterized by relative resistance to chemotherapy. Despite the improvements in the local control rate of limb STS treated with a multidisciplinary approach, a high frequency of recurrence (together with distant metastases) remains a significant problem, particularly in patients with high risk STS (i.e., deep location, large size, recurrent disease, high histological grade) [52].

Up to now ECT of STS has been limited to skin and subcutaneous lesions. In fact, current technology (i.e., the length of the needle electrodes) does not allow the treatment of deep-seated (>3 cm from the skin) or large (indicatively >3 cm) tumors. Nonetheless, the clinical experience with superficial or skin-infiltrating soft tissue sarcomas demonstrates that ECT is able to effectively eradicate all the tumor cells in the treated area. Based on these favorable preliminary findings, an ongoing clinical trial, which was activated in 2009 at the Veneto Region Oncology Research Institute of Padova, is exploring the feasibility and safety, together with antitumor activity, of ECT performed by means of new electrodes on large and/or deep-seated soft tissue tumors. The rationale of this study relies on the hypothesis that longer electrodes and their “custom-made” insertion may generate an electric field tailored to the tumor mass and its margins, thus obtaining a higher local response and tumor control even on large/deep tumors with a single treatment.

Therefore, at the Sarcoma and Melanoma Unit of Padova, a clinical trial on soft tissue tumors is ongoing in order to extend ECT indications, to the patients with large and deep-seated cancers. The study was approved by the Ethics Committee of the University of Padova on 13/07/2009 as ESOPE II Protocol—Electrochemotherapy for deep-seated and large soft tissue tumors.

**Fig. 7:**
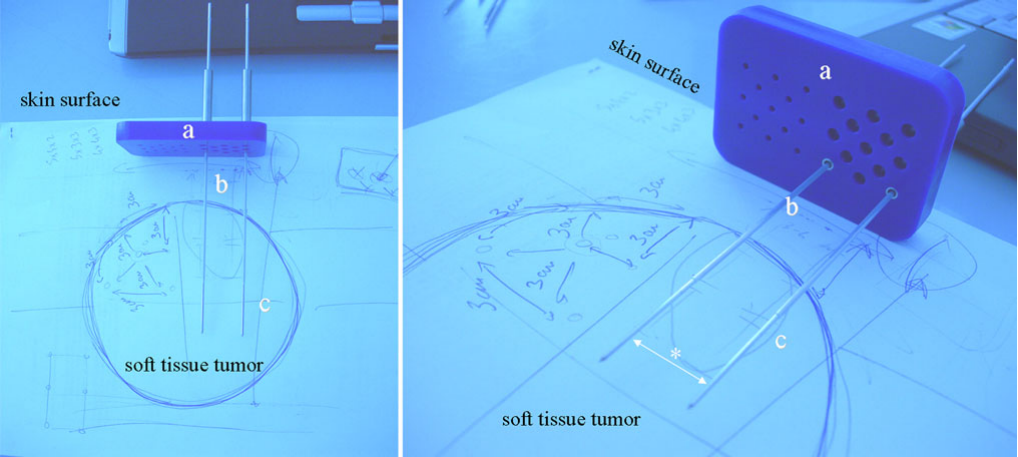
Soft tissue sarcomas, representative model of the electrochemotherapy treatment of a deep soft tissue tumor by means of a plastic stabilization holder (**a**) that is fixed on the patient’s skin surface and has several holes through which long needle electrodes (**b**, **c**) can be inserted percutaneously and thus held parallel. The electrodes consist from insolated (**b**) and active (**c**) part. Their arrangement (i.e., distance in between, *asterisk*) depends on the choice of the operator, based on the data of the pre-treatment planning (colour figure online)

This study is enrolling patients with measurable soft tissue tumor masses of any histotypes who have exhausted, or have been judged unsuitable for conventional treatments. The inclusion criteria were set to require that tumor is between 3 and 6 cm in size or >3 cm in depth. More precisely, the patients enrolled in this study are being treated by means of percutaneously inserted long needle electrodes, connected with the pulse generator Cliniporator VITAE (Igea, Carpi, Italy) that enables clinicians to tailor the electric field, at least within some limits, to the tumor size and shape. In fact, contrary to the standard generator devices for clinical ECT, each needle electrode can be inserted separately, rigorously in a parallel fashion (Fig. 7a), but in a free configuration that can vary according to the tumor shape, the only restriction being the maximum distance between any two electrodes (i.e., 3 cm) (Fig. 7). A plastic needle stabilization holder was custom-designed to help clinician to place the electrodes through the skin and to maintain their planned configuration during the delivery of the electric pulses (Fig. 7). This is particularly important when muscles stimulation occurs because the resulting contraction could displace the inserted needles during the procedure. The new electrodes are configured as 20-cm long, 1.2-mm diameter, partially insulated, metal needles. They are made of stainless steel and the central body is sheathed with an insulating layer to allow the application of the electric field limited to the terminal “active” part (available lengths 20, 30 and 40 mm) and preserve the healthy surrounding tissues. Their greater length and the possibility to be placed at different depths into the tumor, in order to obtain a complete electroporation of the target volume, make these long needle electrodes particularly suitable for ECT of large tissue masses (Fig. 7). In the ongoing trial, after the completion of the feasibility phase (refinement of the operating procedure in the first three patients), the preliminary results after 16 ECT procedures are promising either in terms of patients tolerability (all the treatments were performed under mild general anesthesia), toxicity (mainly skin/soft tissue), and local tumor response. Eleven out of 16 treated patients reported a clinical and radiological complete tumor response on the target lesion.

The implementation of such preliminary data in the ongoing and other future clinical protocols will allow investigators to test the enormous potential of the ECT treatment for STS. The implementation of ECT (together with established high activity and minimal invasiveness) as a treatment option for the management of large and deep-seated tumors such as STS could benefit several patients who would otherwise face burdensome surgery, extreme quality of life loss and reduction in their performance status [28]. Indeed, soft tissue metastases, especially those in skeletal muscle, are frequently painful and ECT may represent a favorable minimally invasive alternative to wide surgical resection, at least in a subgroup of sarcoma patients.

The new electrodes, specific method of their placement and pulse application required some changes in the standard operating procedures of ECT. First, an accurate pre-treatment planning for electrode positioning before the procedure is mandatory for the electroporation of such large cancers. In this way, the electric field distribution around and within the tumor is modeled before each procedure to ensure an electric field amplitude sufficient to cover the whole target volume. The pre-treatment planning is generally focused on keeping the number of electrodes to be inserted at a minimum, so that the procedure is simple and minimally invasive for patients. Further, a radiologic assistance, generally by means of ultrasound scan, is required in the operating room to place the electrodes at the tumor site in a safe and accurate manner. At present, the new procedure seems more time- and technical-demanding than standard ECT and several technical aspects need to be elucidated, in particular the optimization of the pre-treatment planning for electrode insertion and its effective application at the bed side.

### Early clinical experience of treating internal tumors: brain tumors and metastases

Brain metastases are steadily increasing in incidence, both due to rising cancer incidence, and due to better treatments for peripheral disease, leading to brain metastases becoming a more dominant problem. In addition to this, primary brain tumors continue to be associated with considerable morbidity and mortality, and new treatment approaches are warranted. Finally, gene therapy to the brain may also be envisaged, in particular for Parkinson’s disease. The expandable electrode mentioned earlier (Fig. 4) would potentially work for soft tissues on a larger scope, but is particularly well suited for electroporation-based treatments in the brain, since the single point insertion is an important advantage for this application.

Predefined geometry of electrodes predominantly developed for treating brain metastases has been carefully designed and its geometry optimized (Fig. 4). The basic concept is to have one insertion channel, and then to be able to expand electrodes to encompass the target area. The electrode design has been optimized for covering the target volume, by applying volume definitions used in radiotherapy, and defining the most robust design to achieve the intended coverage in the volume [35]. Also, a preclinical version of the electrode has been designed, and tested in a rat brain tumor model [3], showing highly efficient tumor treatment, verified by MRI definition of tumors before randomization to treatment or control, along with follow-up MRI and subsequently histological assessment.

In a phase I clinical trial (NCT01322100), the first patient with brain metastasis was recently treated. The electrode is mounted on a 3D stereotactic frame, and may be positioned very exactly according to *X*, *Y*, *Z* coordinates based on a CT-scan, through a cranial burr hole. When the probe is in position, the electrodes may be deployed to cover the desired target volume. Patients are in full anesthesia, relaxated, and after electrode deployment bleomycin is infused intravenously, before the pulsing sequence is activated. After treatment, the electrodes are retracted and the probe withdrawn. Follow-up includes sequential MRI, which is an imaging modality we have also previously investigated in a rat brain tumor electroporation model [36]. In this first clinical trial, treatment was well tolerated in the first patient, and the trial is still accruing.

### Early clinical experience of treating internal tumors: colorectal tumors

The option of minimally invasive or endoscopic ECT in the treatment of gastrointestinal tumors offers a critically important option for patients with colorectal, gastric or esophageal cancer. While the standard of care involves surgery, this is normally only curative for patients diagnosed with early stage disease. Many patients however are diagnosed with advanced disease. A considerable proportion of diagnosed patients are over the age of 70 and may likely have underlying health complications making radical surgical resection like colostomy or esophagectomy more difficult in terms of recovery and overall impact on the patients’ quality of life. An endoscopic outpatient approach initially to relieve symptoms or forestall complications, could potentially aid responsiveness to other more aggressive therapies.

Table 3 presents the data about world-wide incidence and mortality rates of colorectal cancer (CRC) for 2008 [8]. It is estimated that in North America, Europe and Japan over 550,000 patients are diagnosed with CRC annually. CRC is considered cured in the absence of a recurrence within the first 5 years. Five-year survival rates associated with CRC are as high as 90 % in early-stage disease, and 40–60 % in late-stage disease. Stage I, II, and III cancers are considered potentially curable. In most cases, stage IV cancer is not curable.

**Table 3:** Colorectal cancer incidence rate 2008 [8]

Market	Incident rate	Mortality rate	Mortality rate (%)
USA	153,881	50,640	33
Canada	23,195	7,801	34
China	221,313	110,486	50
Australia/NZ	15,894	5,651	36
Japan	101,656	43,349	43
Europe	334,029	149,159	45
Russia	55,719	37,911	68
Total	905,687	404,997	45

The EndoVE endoscopic device was developed at Cork Cancer Center at University College Cork in Ireland to allow for an endoscopic cancer electroporation treatment, which can target colorectal, gastric and esophageal cancers using vacuum to capture tumor tissue while applying an electroporative electric pulse to the tumor. Briefly, the electrodes of this applicator are made of stainless steel; the dimensions are 26 mm × 10 mm and can be adjusted as required. The width of the small chamber into which the tissue is sucked by the vacuum (under pressure 200–700 mmHg) is 12 mm and the other two dimensions depend on the size of the electrodes. The treatment is minimally disturbing for patients and is easy to perform in an outpatient setting as per standard colonoscopy procedures carried out under sedation. The device itself is disposable and is designed for single use—after each treatment it is removed from the endoscope and disposed of (Fig. 3).

The treatment of spontaneous canine colorectal cancers demonstrated the EndoVE device to be effective and safe with complete tumor resolution noted in the two obstructive cases treated. All procedures were conducted as a simple colonoscopy type procedure with no adverse side effects.

A phase I/II clinical trial in patients with inoperable colorectal cancer is currently ongoing at Cork Cancer Research Centre, Ireland, (http://clinicaltrials.gov/ct2/show/NCT01172860) with the objective being the assessment of efficacy and safety using the EndoVE system. The procedures have been conducted on an outpatient basis under sedation with six treatment sessions performed to date. Patients were treated with bleomycin delivered intravenously in all cases following the electroporation protocols established in the ESOPE study. No adverse events or complications have been noted with excellent tumor regression being noted via MRI and endoscopy visualization. A biopsy assessment of the first patient taken 6 months after initial treatment indicated no detectable tumor tissue. Modifications to the design of the device are currently ongoing based on clinical feedback and a phase I study for esophageal cancer is being prepared.

## Conclusions

Electrochemotherapy (ECT), a combination of high voltage electric pulses and of an anticancer drug, is a highly effective method for local treatment of tumors. It has achieved great results in treatment of cutaneous and subcutaneous tumors. In order to be able to use ECT also for treatment of internal tumors (in particular in treating liver metastasis or esophageal tumors due to proximity of the heart), the treatment must be performed safely, e.g., without interfering with heart electrical activity [17, 37], while providing appropriate and effective electroporative electric pulses to the whole tumor mass, thus effectively eradicating tumor mass. Further advancement of ECT is not without hurdles, however, and some of them are already being addressed by the described technological innovations. To advance ECT from treating cutaneous and subcutaneous tumors to treating internal solid tumors, new technological developments are needed that enable treatment of these relatively difficult to reach internal tumors. Treatment of such tumors is made possible by development of new clinical devices that allow for pulse delivery to larger number of electrodes, by design of new types of electrodes suited for specific application areas and by introduction of synchronization of pulse delivery with ECG, which should be mandatory whenever the treatment site is located in vicinity of the heart (e.g., liver metastases). To increase the probability of complete tumor eradication, the electrodes have to be accurately positioned, first to provide an adequate extent of electroporation of all tumor cells and second to prevent or minimize the damage induced in critical healthy tissues or organs in vicinity of the treatment area. This can be achieved by: (a) patient-specific numerical treatment planning, and (b) a real-time image-guided electrode insertion that enables accurate positioning of electrodes according to treatment plan, for either fixed or variable electrode geometries. A lot of effort is currently directed towards further improvement in these two areas. The requirement for percutaneous treatment will foster further technical development for treatment of abdominal tumors; a laparoscopic approach, that will necessitate adapting and using image guidance navigation systems. Further steps are also needed in actual monitoring of tissue electroporation [36, 73] and electric field distribution [33, 34] which will allow better control of electrode positioning and successful treatment even in a difficult to reach locations.

## References

[1] Aaronson NK, Ahmedzai S, Bergman B, Bullinger M, Cull A, Duez NJ, Filiberti A, Flechtner H, Fleishman SB, de Haes JC (1993). The European Organization for Research and Treatment of Cancer QLQ-C30: a quality-of-life instrument for use in international clinical trials in oncology. J Natl Cancer Inst.

[2] Abdalla EK, Vauthey J-N, Ellis LM, Ellis V, Pollock R, Broglio KR, Hess K, Curley SA (2004). Recurrence and outcomes following hepatic resection, radiofrequency ablation, and combined resection/ablation for colorectal liver metastases. Ann Surg.

[3] Agerholm-Larsen B, Iversen HK, Ibsen P, Moller JM, Mahmood F, Jensen KS, Gehl J (2011). Preclinical validation of electrochemotherapy as an effective treatment for brain tumors. Cancer Res.

[4] Belehradek M, Domenge C, Luboinski B, Orlowski S, Belehradek J, Mir LM (1993). Electrochemotherapy, a new antitumor treatment—1st clinical phase I-II trial. Cancer.

[5] Bertacchini C, Margotti PM, Bergamini E, Lodi A, Ronchetti M, Cadossi R (2007). Design of an irreversible electroporation system for clinical use. Technol Cancer Res Treat.

[6] Bhardwaj N, Strickland AD, Ahmad F, Dennison AR, Lloyd DM (2010). Liver ablation techniques: a review. Surg Endosc.

[7] Bianchi G, Campanacci L, Fini M (2010) Electrochemotherapy for the treatment of osteolytic bone metastasis: a phase I/II clinical trial. EMSOS 2010 Annual Meeting, 5–7 May 2010, Birmingham, UK

[8] Bray F, Ren J-S, Masuyer E, Ferlay J (2012). Global estimates of cancer prevalence for 27 sites in the adult population in 2008. Int J Cancer.

[9] Callstrom MR, Charboneau JW, Goetz MP, Rubin J, Atwell TD, Farrell MA, Welch TJ, Maus TP (2006). Image-guided ablation of painful metastatic bone tumors: a new and effective approach to a difficult problem. Skeletal Radiol.

[10] Campana LG, Mocellin S, Basso M, Puccetti O, De Salvo GL, Chiarion-Sileni V, Vecchiato A, Corti L, Rossi CR, Nitti D (2009). Bleomycin-based electrochemotherapy: clinical outcome from a single institution’s experience with 52 patients. Ann Surg Oncol.

[11] Cemazar M, Milacic R, Miklavcic D, Dolzan V, Sersa G (1998). Intratumoral cisplatin administration in electrochemotherapy: antitumor effectiveness, sequence dependence and platinum content. Anticancer Drugs.

[12] Coleman RE (2001). Metastatic bone disease: clinical features, pathophysiology and treatment strategies. Cancer Treat Rev.

[13] Colombo GL, Matteo SD, Mir LM (2008). Cost-effectiveness analysis of electrochemotherapy with the Cliniporatortrade mark vs other methods for the control and treatment of cutaneous and subcutaneous tumors. Ther Clin Risk Manag.

[14] Corovic S, Al Sakere B, Haddad V, Miklavcic D, Mir LM (2008). Importance of contact surface between electrodes and treated tissue in electrochemotherapy. Technol Cancer Res Treat.

[15] Corovic S, Zupanic A, Miklavcic D (2008). Numerical modeling and optimization of electric field distribution in subcutaneous tumor treated with electrochemotherapy using needle electrodes. IEEE Trans Plasma Sci.

[16] Daud AI, DeConti RC, Andrews S (2008). Phase I trial of interleukin-12 plasmid electroporation in patients with metastatic melanoma. J Clin Oncol.

[17] Deodhar A, Dickfeld T, Single GW, Hamilton WC, Thornton RH, Sofocleous CT, Maybody M, Gónen M, Rubinsky B, Solomon SB (2011). Irreversible electroporation near the heart: ventricular arrhythmias can be prevented with ECG synchronization. Am J Roentgenol.

[18] Derogar M, Orsini N, Sadr-Azodi O, Lagergren P (2012). Influence of major postoperative complications on health-related quality of life among long-term survivors of esophageal cancer surgery. J Clin Oncol.

[19] Domenge C, Orlowski S, Luboinski B, De Baere T, Schwaab G, Belehradek J, Mir LM (1996). Antitumor electrochemotherapy: new advances in the clinical protocol. Cancer.

[20] Edhemovic I, Gadzijev EM, Brecelj E (2011). Electrochemotherapy: a new technological approach in treatment of metastases in the liver. Technol Cancer Res Treat.

[21] Ferlay J, Parkin DM, Steliarova-Foucher E (2010). Estimates of cancer incidence and mortality in Europe in 2008. Eur J Cancer.

[22] Fini M, Tschon M, Ronchetti M, Cavani F, Bianchi G, Mercuri M, Alberghini M, Cadossi R (2010). Ablation of bone cells by electroporation. J Bone Jt Surg Br.

[23] Front D, Israel O, Iosilevsky G, Even-Sapir E, Ben-Haim S, Frenkel A, Ber R, Milstein D, Kolodny GM (1990). Administered dose and tumor dose of bleomycin labeled with cobalt-57 in mice and men. J Nucl Med.

[24] Gatta G, Capocaccia R, Sant M (2000). Understanding variations in survival for colorectal cancer in Europe: a EUROCARE high resolution study. Gut.

[25] Gehl J, Geertsen PF (2006). Palliation of haemorrhaging and ulcerated cutaneous tumours using electrochemotherapy. Eur J Cancer Suppl.

[26] Gehl J, Videbaek K (2009) Electrode introducer device. Patent EP2032057

[27] Gothelf A, Mir LM, Gehl J (2003). Electrochemotherapy: results of cancer treatment using enhanced delivery of bleomycin by electroporation. Cancer Treat Rev.

[28] Griesser MJ, Gillette B, Crist M, Pan X, Muscarella P, Scharschmidt T, Mayerson J (2012). Internal and external hemipelvectomy or flail hip in patients with sarcomas: quality-of-life and functional outcomes. Am J Phys Med Rehabil.

[29] Hompes D, Prevoo W, Ruers T (2011). Radiofrequency ablation as a treatment tool for liver metastases of colorectal origin. Cancer Imaging.

[30] Jarm T, Cemazar M, Miklavcic D, Sersa G (2010). Antivascular effects of electrochemotherapy: implications in treatment of bleeding metastases. Expert Rev Anticancer Ther.

[31] Jeremic B, Shibamoto Y, Acimovic L, Milicic B, Milisavljevic S, Nikolic N, Aleksandrovic J, Igrutinovic I (1998). A randomized trial of three single-dose radiation therapy regimens in the treatment of metastatic bone pain. Int J Radiat Oncol Biol Phys.

[32] Kotnik T, Pucihar G, Miklavcic D (2010). Induced transmembrane voltage and its correlation with electroporation-mediated molecular transport. J Membr Biol.

[33] Kranjc M, Bajd F, Sersa I, Miklavcic D (2011). Magnetic resonance electrical impedance tomography for monitoring electric field distribution during tissue electroporation. IEEE Trans Med Imaging.

[34] Kranjc M, Bajd F, Sersa I, Woo EJ, Miklavcic D (2012). Ex vivo and in silico feasibility study of monitoring electric field distribution in tissue during electroporation based treatments. PLoS One.

[35] Mahmood F, Gehl J (2011). Optimizing clinical performance and geometrical robustness of a new electrode device for intracranial tumor electroporation. Bioelectrochemistry.

[36] Mahmood F, Hansen RH, Agerholm-Larsen B, Jensen KS, Iversen HK, Gehl J (2011). Diffusion-weighted MRI for verification of electroporation-based treatments. J Membr Biol.

[37] Mali B, Jarm T, Corovic S, Paulin-Kosir MS, Cemazar M, Sersa G, Miklavcic D (2008). The effect of electroporation pulses on functioning of the heart. Med Biol Eng Comput.

[38] Mali B, Jarm T, Gadzijev E, Sersa G, Miklavcic D, Bamidis PD, Pallikarakis N (2010). Changes in electrocardiogram during intra-abdominal electrochemotherapy: a preliminary report. XII Mediteranean Conference on Medical and Biological Engineering and computing 2010, MEDICON 2010, IFMBE Proceedings 29.

[39] Mali B, Jarm T, Snoj M, Sersa G, Miklavcic D (2012) Antitumor effectiveness of electrochemotherapy: A systematic review and meta-analysis. Eur J Surg Oncol. doi:10.1016/j.ejso.2012.08.016 10.1016/j.ejso.2012.08.016 22980492

[40] Manfredi S, Lepage C, Hatem C, Coatmeur O, Faivre J, Bouvier A-M (2006). Epidemiology and management of liver metastases from colorectal cancer. Ann Surg.

[41] Marty M, Sersa G, Garbay JR (2006). Electrochemotherapy—an easy, highly effective and safe treatment of cutaneous and subcutaneous metastases: results of ESOPE (European Standard Operating Procedures of Electrochemotherapy) study. Eur J Cancer Suppl.

[42] Matthiessen LW, Chalmers RL, Sainsbury DCG, Veeramani S, Kessell G, Humphreys AC, Bond JE, Muir T, Gehl J (2011). Management of cutaneous metastases using electrochemotherapy. Acta Oncol.

[43] Matthiessen LW, Johannesen HH, Hendel HW, Moss T, Kamby C, Gehl J (2012). Electrochemotherapy for large cutaneous recurrence of breast cancer: a phase II clinical trial. Acta Oncol.

[44] Miklavcic D, Beravs K, Semrov D, Cemazar M, Demsar F, Sersa G (1998). The importance of electric field distribution for effective in vivo electroporation of tissues. Biophys J.

[45] Miklavcic D, Corovic S, Pucihar G, Pavselj N (2006). Importance of tumour coverage by sufficiently high local electric field for effective electrochemotherapy. Eur J Cancer Suppl.

[46] Miklavcic D, Snoj M, Zupanic A, Kos B, Cemazar M, Kropivnik M, Bracko M, Pecnik T, Gadzijev E, Sersa G (2010). Towards treatment planning and treatment of deep-seated solid tumors by electrochemotherapy. Biomed Eng Online.

[47] Mir LM, Banoun H, Paoletti C (1988). Introduction of definite amounts of nonpermeant molecules into living cells after electropermeabilization: direct access to the cytosol. Exp Cell Res.

[48] Mir LM, Gehl J, Sersa G, Collins CG, Garbay JR, Billard V, Geertsen PF, Rudolf Z, O’Sullivan GC, Marty M (2006). Standard operating procedures of the electrochemotherapy: instructions for the use of bleomycin or cisplatin administered either systemically or locally and electric pulses delivered by the Cliniporator™ by means of invasive or non-invasive electrodes. Eur J Cancer Suppl.

[49] Mir LM, Glass LF, Sersa G (1998). Effective treatment of cutaneous and subcutaneous malignant tumours by electrochemotherapy. Br J Cancer.

[50] Mir LM, Orlowski S, Belehradek J, Teissié J, Rols MP, Sersa G, Miklavcic D, Gilbert R, Heller R (1995). Biomedical applications of electric pulses with special emphasis on antitumor electrochemotherapy. Bioelectrochemistry Bioenerg.

[51] Morales-de la Peña M, Elez-Martínez P, Martín-Belloso O (2011). Food preservation by pulsed electric fields: an engineering perspective. Food Eng Rev.

[52] O’Sullivan B, Pisters PW (2003). Staging and prognostic factor evaluation in soft tissue sarcoma. Surg Oncol Clin N Am.

[53] Orlowski S, Belehradek J, Paoletti C, Mir LM (1988). Transient electropermeabilization of cells in culture. Increase of the cytotoxicity of anticancer drugs. Biochem Pharmacol.

[54] Pathak S, Jones R, Tang JM, Parmar C, Fenwick S, Malik H, Poston G (2011). Ablative therapies for colorectal liver metastases: a systematic review. Colorectal Dis.

[55] Pavliha D, Kos B, Zupanic A, Marcan M, Sersa G, Miklavcic D (2012). Patient-specific treatment planning of electrochemotherapy: procedure design and possible pitfalls. Bioelectrochemistry.

[56] Pavselj N, Bregar Z, Cukjati D, Batiuskaite D, Mir LM, Miklavcic D (2005). The course of tissue permeabilization studied on a mathematical model of a subcutaneous tumor in small animals. IEEE Trans Biomed Eng.

[57] Pavselj N, Miklavcic D (2008). Numerical modeling in electroporation-based biomedical applications. Radiol Oncol.

[58] Pucihar G, Krmelj J, Rebersek M, Napotnik TB, Miklavcic D (2011). Equivalent pulse parameters for electroporation. IEEE Trans Biomed Eng.

[59] Quaglino P, Mortera C, Osella-Abate S, Barberis M, Illengo M, Rissone M, Savoia P, Bernengo MG (2008). Electrochemotherapy with intravenous bleomycin in the local treatment of skin melanoma metastases. Ann Surg Oncol.

[60] Sack M, Sigler J, Frenzel S, Eing C, Arnold J, Michelberger T, Frey W, Attmann F, Stukenbrock L, Müller G (2010). Research on industrial-scale electroporation devices fostering the extraction of substances from biological tissue. Food Eng Rev.

[61] Sardesai NY, Weiner DB (2011). Electroporation delivery of DNA vaccines: prospects for success. Curr Opin Immunol.

[62] Sersa G (2006). The state-of-the-art of electrochemotherapy before the ESOPE study; advantages and clinical uses. Eur J Cancer Suppl.

[63] Sersa G, Cemazar M, Miklavcic D (1995). Antitumor effectiveness of electrochemotherapy with cis-diamminedichloroplatinum(II) in mice. Cancer Res.

[64] Sersa G, Cufer T, Paulin SM, Cemazar M, Snoj M (2012). Electrochemotherapy of chest wall breast cancer recurrence. Cancer Treat Rev.

[65] Sersa G, Jarm T, Kotnik T (2008). Vascular disrupting action of electroporation and electrochemotherapy with bleomycin in murine sarcoma. Br J Cancer.

[66] Sersa G, Miklavcic D, Cemazar M, Rudolf Z, Pucihar G, Snoj M (2008). Electrochemotherapy in treatment of tumours. Eur J Surg Oncol.

[67] Sersa G, Stabuc B, Cemazar M, Jancar B, Miklavcic D, Rudolf Z (1998). Electrochemotherapy with cisplatin: potentiation of local cisplatin antitumour effectiveness by application of electric pulses in cancer patients. Eur J Cancer.

[68] Sersa G, Stabuc B, Cemazar M, Miklavcic D, Rudolf Z (2000). Electrochemotherapy with cisplatin: clinical experience in malignant melanoma patients. Clin Cancer Res.

[69] Teissie J, Golzio M, Rols MP (2005). Mechanisms of cell membrane electropermeabilization: a minireview of our present (lack of ?) knowledge. Biochim Biophys Acta.

[70] Testori A, Rutkowski P, Marsden J, Bastholt L, Chiarion-Sileni V, Hauschild A, Eggermont AMM (2009). Surgery and radiotherapy in the treatment of cutaneous melanoma. Ann Oncol.

[71] Testori A, Tosti G, Martinoli C (2010). Electrochemotherapy for cutaneous and subcutaneous tumor lesions: a novel therapeutic approach. Dermatol Ther.

[72] Toepfl S, Heinz V, Knorr D (2007). High intensity pulsed electric fields applied for food preservation. Chem Eng Process.

[73] Zhang Y, Guo Y, Ragin AB, Lewandowski RJ, Yang G-Y, Nijm GM, Sahakian AV, Omary RA, Larson AC (2010). MR imaging to assess immediate response to irreversible electroporation for targeted ablation of liver tissues: preclinical feasibility studies in a rodent model. Radiology.

[74] Zupanic A, Corovic S, Miklavcic D (2008). Optimization of electrode position and electric pulse amplitude in electrochemotherapy. Radiol Oncol.

